# Communication Bridge 2: Primary and secondary outcomes from a global telehealth randomized controlled trial for persons with primary progressive aphasia and their communication partners

**DOI:** 10.1002/alz.095391

**Published:** 2025-01-09

**Authors:** Emily J Rogalski, Alfred Rademaker, Aimee Mooney, Melanie Fried‐Oken, Matthew Bona, Angela C Roberts

**Affiliations:** ^1^ University of Chicago, Chicago, IL USA; ^2^ Northwestern University, Chicago, IL USA; ^3^ Oregon Health and Science University, Portland, OR USA; ^4^ OHSU, Portland, OR USA; ^5^ Northwestern University, Evanston, IL USA; ^6^ Western University, London, ON Canada

## Abstract

**Background:**

Primary progressive aphasia (PPA) is a currently incurable and relatively rare language‐based neurodegenerative dementia syndrome, which negatively impacts communication and quality of life. Non‐pharmacologic interventions show promise but have lacked randomized controlled trials (RCT). Here we report outcomes for Communication Bridge‐2 (CB2), the first global telemedicine speech‐language (RCT) for PPA.

**Method:**

CB2 is an international, single enrollment site, Phase 2, Stage 2, parallel‐group, active control, behavioral RCT delivered via video‐chat to individuals with PPA and their communication partners. CB2 is supported by a custom web application. Participants were randomized 3:2 into one of two arms: Communication Bridge™ a dyadic intervention based on communication participation models, or, the Control intervention, a non‐dyadic intervention based on impairment models. Participants completed two intervention blocks over ∼12 months. Primary outcomes included communication confidence and participation measures assessed at baseline, each intervention block, and ∼12 months post‐enrollment. Other outcomes included word and script performance. Post‐study interviews captured participant experiences and informed feasibility.

**Result:**

Ninety‐five PPA participant dyads (mean enrollment age of PPA participants: 67.1 years, 49% female) were randomized (n = 4 countries). Dropout was <10%. Procedural and documentation fidelity were high (>90%, for both). Theoretical fidelity showed strong within‐arm alignment. Experimental arm superiority was demonstrated at Block 1 for the Goal Attainment Scale, measurement, a primary participation outcome evaluated by a blinded clinician. The Communicative Participation Item Bank showed positive within‐group responsiveness at Block 1 exclusively for the experimental arm. Communication confidence measurement was less responsive. Other outcomes including word and script training, showed significant gains at Block 1 for both groups. Post‐study interviews suggested high feasibility.

**Conclusion:**

Results suggest feasibility and initial efficacy for a global telemedicine intervention, providing an exciting path for implementation and dissemination of clinically meaningful interventions for Alzheimer’s and related dementias using telehealth models that can improve care access.

